# Homocysteine aggravates DNA damage by impairing the FA/Brca1 Pathway in NE4C murine neural stem cells

**DOI:** 10.7150/ijms.49246

**Published:** 2020-09-09

**Authors:** Yana Yan, Yandan Yin, Xiaofang Feng, Yuan Chen, Jiamin Shi, Huachun Weng, Dan Wang

**Affiliations:** 1 Department of Pediatrics, The First Affiliated Hospital of Wenzhou Medical University, Wenzhou 325000, Zhejiang, P. R. China.; 2 Department of Pediatrics, Taizhou Women and Children's Hospital of Wenzhou Medical University, Taizhou 318000, Zhejiang, P. R. China.

**Keywords:** homocysteine, DNA damage, Fanconi anemia pathway, Brca1, neural stem cell

## Abstract

There is existing evidence that elevated homocysteine (Hcy) levels are risk factors for some neurodegenerative disorders. The pathogenesis of neurological diseases could be contributed to excessive cell dysfunction and death caused by defective DNA damage response (DDR) and accumulated DNA damage. Hcy is a neurotoxic amino acid and acts as a DNA damage inducer. However, it is not clear whether Hcy participates in the DDR. To investigate the effects of Hcy on DNA damage and the DDR, we employed mitomycin C (MMC) to cause DNA damage in NE4C murine neural stem cells (NSCs). Compared to treatment with MMC alone, we found that co-treatment with MMC and Hcy worsened DNA damage and increased death in NE4C cells. Intriguingly, in this DNA damage model mimicked by MMC, immunoblotting results showed that the monoubiquitination levels of Fanconi anemia complementation group I (Fanci) and Fanconi anemia complementation group D2 (Fancd2) were decreased to about 60.3% and 55.7% by supplementing cell culture medium with Hcy, indicating Hcy inactivates the function of Fanci and Fancd2 in DNA damage conditions. Given Breast Cancer 1 (BRCA1) is an important downstream of FANCD2, we next detected the interaction between Fancd2 and Brca1 in NE4C cells. Compared to treatment with MMC alone, the Fancd2-Brca1 interaction and the amount of Brca1 on chromatin were decreased when cells were co-exposed to MMC and Hcy, suggesting Hcy could impair the Fanconi anemia (FA)/Brca1 pathway. Taken together, our study demonstrates that Hcy may enhance cell death, which contributes to the accumulation of DNA damage and promotion of hypersensitivity to cytotoxicity by impairing the FA/Brca1 pathway in murine NSCs in the presence of DNA damage.

## Introduction

Homocysteine (Hcy) is a non-protein amino acid, formed by methionine (Met) derived from the diet and secreted during metabolism 1. An elevated level of Hcy in the blood results in hyperhomocysteinemia (HHcy) and is closely associated with some neurodegenerative diseases, including dementia, Alzheimer's disease and Parkinson's disease 2-5. The deleterious influences of HHcy have also been reported in the developing central nervous system (CNS) 1,6. Public health strategies suggest that all women of reproductive age supplement their diet with 400 μg of folic acid each day, for the sake of decreasing the risks associated with HHcy, especially with neural tube defects (NTDs) 7. HHcy may diminish methyl donors, decrease DNA methylation, and accumulate DNA damage 8,9. As a DNA damage agent, Hcy can cause several types of DNA damage including DNA strand breaks, interstrand cross-links (ICLs) and mutation 10-12. The main reasons of Hcy-caused DNA damage are considered to be the induction of oxidative stress, disruption of the DNA methylation cycle, and uracil misincorporation 13. A series of data have also been shown that Hcy is a potent neurotoxin, which contributes to oxidative stress in the brain, where it activates N-methyl-D-aspartate (NMDA) receptors and mobilizes intracellular calcium stores leading to caspase activation, DNA damage, and neuronal apoptosis *in vitro* and *vivo* 10,11,14,15. The events in the apoptotic pathway activated by Hcy appear to be ordered as follows: DNA damage, caspase activation, mitochondrial membrane potential decline, and nuclear disintegration 11. Thus, DNA damage is an early event for Hcy neurotoxicity.

Accumulation of DNA damage, the main reason for excessive apoptosis/death of neural cells, is a critical pathological cause of neurological diseases 16-19. During neurodevelopment, the rapid cellular proliferation can lead to replication-induced DNA damage 20. In mature brains, neural cells are also prone to DNA damage caused by metabolites including oxygen free radicals, homocysteine and amyloid β-peptide (Aβ) 4,17,19,21. Failure to effectively overcome DNA damage results in damage accumulation, mitotic catastrophe, chromosomal rearrangements, and cell death. Neural stem cells (NSCs) self-renew, proliferate and differentiate into three major neural cell types (neurons, oligodendrocytes and astrocytes). Due to their intense self-renewal and rapid proliferation, NSCs are susceptible to DNA damage agents 22. Therefore, appropriate activation of the DNA damage response (DDR) to repair damage and maintain the genetic integrity of NSCs may ensure correct brain development and homeostasis.

Interstrand cross-links (ICLs) are severe DNA lesions induced by numerous agents, including mitomycin C (MMC), cisplatin and endogenous products of lipid peroxidation 23,24. By covalently linking both DNA strands, ICLs are highly toxic, as they can physically block DNA melting, transcription, and replication 25. Failure to repair ICLs may cause DNA strand breaks, chromosomal rearrangements, and cell death. To remove ICLs and repair the DNA, Fanconi anemia (FA) proteins are required. Fifteen FA genes have been identified and their protein products are thought to share a common pathway. Following DNA damage, eight FA proteins (A, B, C, E, F, G, L, and G) are assembled into the nuclear FA core complex that monoubiquitylates its two downstream targets, Fanconi Anemia Complementation Group I (FANCI) and Fanconi Anemia Complementation Group D2 (FANCD2), followed by their recruitment to chromatin 26. FANCD2 and FANCI are activated by monoubiquitination, and then organize the pathway to initiate checkpoints and resistance to ICLs 27,28. Moreover, monoubiquitinated FANCD2 (ub-FANCD2) is required for FANCI monoubiquitination (ub-FANCI) and recruitment of Breast Cancer 1 (BRCA1), Fanconi Anemia Complementation Group J (FANCJ), and Fanconi Anemia Complementation Group N (FANCN), to form damage-induced foci on chromatin 29,30. FA patients frequently have brain anomalies, including microcephaly and developmental delay 31. Loss of *Fancd2* in animal models results in DNA damage-induced apoptosis of neural cells during neurogenesis 32,33. Moreover, dysfunction of BRCA1 provokes neural progenitor cell apoptosis and reduces brain size during development and evolution 34. The above data implicate that the FA/BRCA1 pathway is essential for maintaining genetic stability, dysfunction of this pathway may aggravate DNA damage and apoptosis in neural cells.

Previous studies have been shown that Hcy can sensitize cells to DNA damage agents 15,35; however, the precise mechanism by which Hcy exacerbates DNA damage in neural cells is not fully discovered. Mitomycin C (MMC), a common chemotherapeutic drug for many malignancies, inhibits cell proliferation and causes death by inducing ICLs 36-38. Here, we used cultured murine NSCs to test the hypothesis that Hcy may aggravate DNA damage and promote MMC-induced cytotoxicity by impairing the FA/Brca1 pathway in the presence of DNA damage.

## Materials and Methods

### Cell culture, treatment and transfection

Murine NE4C neural stem cells (ATCC ® CRL-2925^™^) were maintained in Minimum Essential Medium (MEM; Gibco, Carlsbad, CA, USA) with 10% fetal bovine serum, 2 mM glutamine, 100 U/ml penicillin, and 100 μg/ml streptomycin. The NE4C cells were treated with saline (control), 0.1 μM MMC (Sigma-Aldrich, St Louis, MO, USA), or a combination of 0.1 μM MMC and 0.5 mM Hcy (Sigma-Aldrich) for 24 h as previously described (35). MMC and Hcy were dissolved in saline. The cells were transfected with plasmids using Lipofectamine 2000 (Invitrogen, Carlsbad, CA, USA) according to the manufacturer's protocol for 48 h. The Fancd2 and Brca1 open reading frame plasmids were purchased from Origene (Frederick, MD, USA).

### Reverse transcription and quantitative real-time PCR (qPCR)

Total RNA was extracted from cell by TRIzol® Reagent (Invitrogen) according to the manufacturer's instructions. For qPCR, each sample was run in triplicate in a 20 μl reaction with 250 nM forward and reverse primers (Sangon, Shanghai, China), 10 μl of SYBR Green Supermix (Bio-Rad, Berkeley, CA, USA) and 10 ng of cDNA. The qPCR conditions were as follows: initial denaturation at 95 °C for 5 min, followed by 35 cycles of amplification (denaturation at 95 °C for 30 sec, annealing at 60 °C for 30 sec, extension the clones of *Fancd2*, *Fanci* and *Gapdh* at 72 °C for 4.5, 4 and 1min, respectively). *Gapdh* was used as a control. Reactions were performed in the BIO-RAD CFX Real-Time System (Bio-Rad, Berkeley, CA, USA) and relative mRNA expression levels were calculated using the ΔCt method (2^-ΔΔCt^). Primers are listed in **Table 1.**

### Cell lysis, western blot and co-immunoprecipitation

For western blot (WB), cells were lysed with 0.5% NP40 buffer containing protease inhibitor cocktail (Sigma-Aldrich) on ice. Protein samples were separated by 4-15% gradient gels (Bio-Rad) and transferred to nitrocellulose membranes. After blocking with 5% skimmed milk for 1 h, membranes were incubated with the following primary antibodies: Fancd2 (1:1000), Fanci (1:2000), H2ax (1:1000), γ-H2ax (1:1000), Flag (1:3000) and Myc (1:3000). Blots were visualized using an ECL Plus system (Thermo, Waltham, MA, USA). The line densitometry was quantified by ImageJ software (version 1.61, National Institutes of Health, Bethesda, MD, USA).

For co-immunoprecipitation (co-IP), cells were lysed with 0.1% NP40 buffer containing protease inhibitor cocktail. Cell lysates were incubated with Flag-beads and rotated for 2 h at 4 °C. Immunoprecipitation beads were washed three times with 0.1% NP40 buffer. Washed IP beads were boiled in 1× loading buffer and detected for bound proteins by WB.

### Detection of DNA damage

DNA damage was detected by Comet assay kit (Trevigen, Gaithersburg, MD, USA) as described previously 39. After harvest, NE4C cells were embedded into 1% low melting point agarose on microscope slides. Following treatment with chilled lysis buffer at 4 °C overnight, the slides were immersed into alkaline buffer and allowed DNA to unwind and denature at room temperature for 20 min. Then slides were transferred to an electrophoresis unit with ice-cold alkaline running buffer, and electrophoresis was conducted at 250 mA for 30 min. After electrophoresis, slides were stained with SYBR green (Sigma-Aldrich) and analyzed by using a Leica DMI 4000B epifluorescence microscope and the CASP software. Nuclei with damaged DNA appear as a comet with a bright head and a tail, while nuclei with undamaged DNA appear round without a tail. The percentages of comet-positive cells and Olive tail movement (OTM) were used to assess DNA damage 39.

### Cell viability assay

To evaluate cell viability, the Cell Counting Kit-8 (CCK8) assay (Dojindo Laboratories, Mashikimachi, Kumamoto, Japan) was used. NEC4 cells were seeded in 96-well plates at the initial cell concentration of 4 × 10^3^ cells/well. Following treatment with MMC or MMC+Hcy for 24 h, cells were added with 10 μl CCK8 and incubated for 2 h at 37 °C. Optical density (OD) value at 450 nm was acquired using a microplate reader (BioTek, Winusky, VT, USA).

### Flow cytometry

After treatment with MMC or MMC+Hcy for 24 h, cells were harvested and resuspended in 1× binding buffer at a concentration of 1 × 10^6^ cells/ml. Then, cells were stained with 5 μl FITC-annexin V and 5 μl PI (B.D., San Diego, CA, USA) for 15 min at room temperature in the dark. Flow cytometric analysis was performed on Accuri C6 (B.D.) and analyzed using FlowJo7.6.2 (Tree Star, San Carlos, CA, USA).

### Caspase-3 activity assay

Caspase-3 activity assay kit (Cell Signaling Technology, Danvers, MA, USA) was used according to the manufacture's introductions. Harvested NE4C cells were washed with ice-cold phosphate-buffered saline followed by the addition of cell lysis buffer. Cell lysate was added to assay plates containing the substrate solution, and plates were incubated at 37 °C in the dark for 2 h. Fluorescence was measured (excitation, 380 nm; emission, 460 nm) and expressed in relative fluorescence units.

### Statistical analysis

Statistical analysis was performed using SPSS version 22.0 (SPSS Inc., Chicago, IL, USA). Results were presented as mean ± standard deviation (SD). One-way analysis of variance (ANOVA) were used for the detection of statistical significance. A *P* value < 0.05 was considered statistically significant.

## Results

### Hcy enhances the cytotoxicity of MMC in NE4C cells

**Figure 1:**
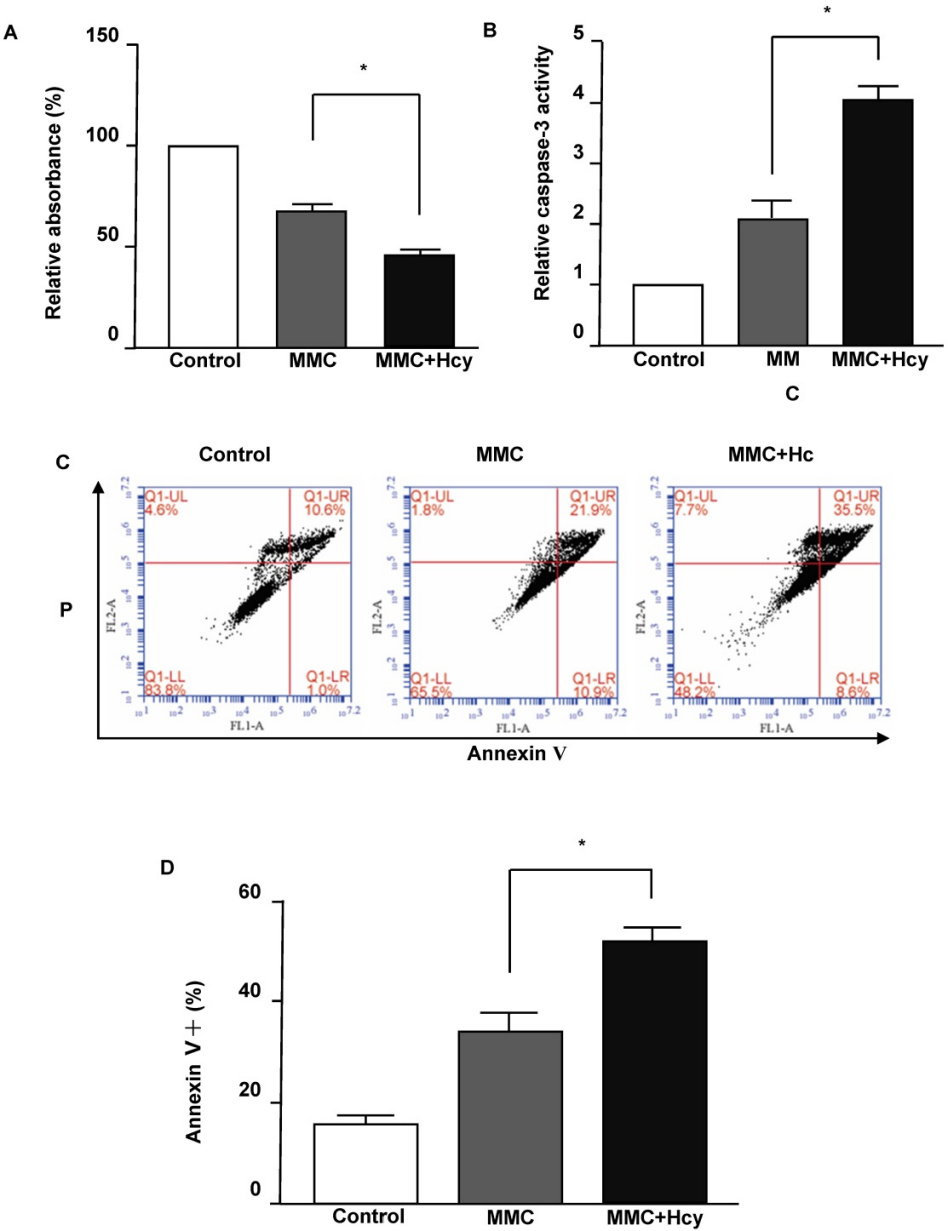
** Hcy enhances the cytotoxity of MMC in murine neural stem cells.** (**A**) Survival of NE4C cells is detected by the CCK8 assay and quantified by the relative absorbance. (**B**) Caspase-3 activity was measured using the Caspase-3 Activity Assay Kit. (**C**) Representative apoptosis dot-plots of the mentioned interventions. (**D**) Representative statistical results of Annexin V positive and propidium (PI) positive cells (%). Values are shown as mean ± SD of six independent experiments. *, *P* < 0.05, **, *P* < 0.01. Control: cells treated without MMC or Hcy.

To investigate the effects of Hcy on inhibiting cell viability under DNA damage, the NE4C cells were exposed to MMC with or without Hcy for 24 h. The Cell Counting Kit-8 (CCK8) assay displayed that cells treated with MMC alone had a survival of 68.31 ± 7.78% (**Fig. 1A**). By contrast, the combined treatment of MMC and Hcy reduced the survival to 45.12 ± 7.62%. To detect whether the cell death induced by combined treatment of Hcy and MMC was due to apoptosis, we evaluated the caspase-3 activity, which is a marker of apoptosis. The level of caspase-3 activity in cells co-treated with MMC and Hcy was higher than that in cells treated with MMC alone (**Fig. 1B**). We also assessed the effects of Hcy on NE4C cell apoptosis using annexin V/propidium iodide staining following MMC exposure (**Fig. 1C and D**). Compared with cells treated with MMC alone (33.60 ± 7.19%), the percentage of dead cells was 51.73 ± 4.60% when cells were co-exposed to Hcy and MMC (*P* < 0.01). Together, these results indicate that Hcy remarkably promotes the cytotoxity of MMC in NSCs.

### Hcy aggravates murine NSCs DNA damage induced by MMC

**Figure 2:**
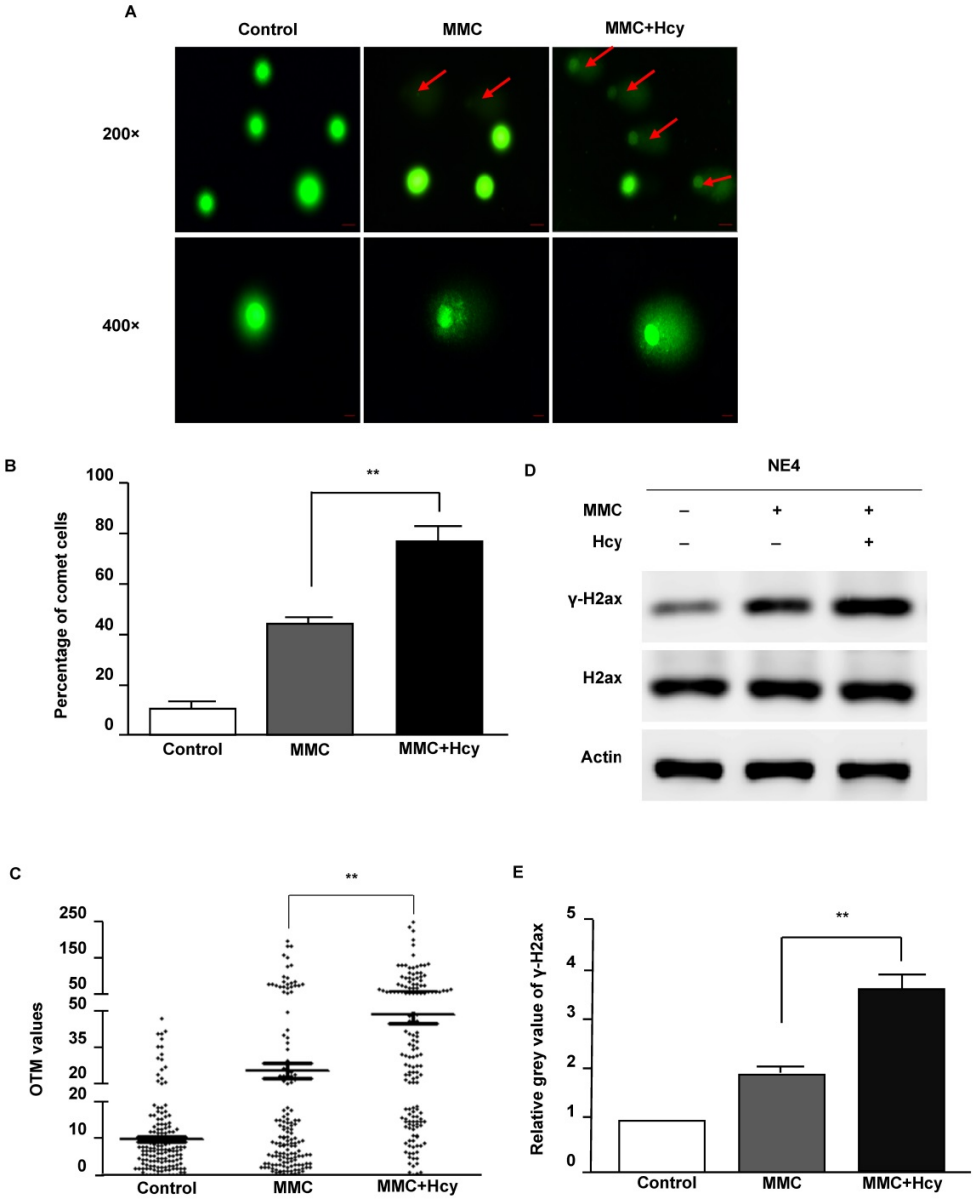
** Hcy aggravates NE4C murine neural stem cell DNA damage induced by MMC.** (**A**) Cultures were exposed to saline (control), 0.1 µM MMC (MMC), or a combination of 0.1 µM MMC and 0.5 mM Hcy (MMC+Hcy) for 24 h. The alkaline Comet assay was conducted in NE4C murine neural stem cells (NSCs). DNA was stained with SYBR Green. The scale bars are 20 µm and 10 µm at 200 × and 400 × magnification, respectively. Red arrow indicates a comet cell. (**B**) Total green cells were counted and the percentage of comet cells was calculated and plotted. (**C**) Images (400 ×) of 50 cells per slide for a total of 150 cells per sample were randomly opted and analyzed for Olive tail movement (OTM) using CASP software. The middle horizontal line, mean value of each sample. (**D**) Western blot (WB) analysis of treated NSCs with γ-H2ax and H2ax antibodies. (**E**) Representative WB statistical results are shown (n = 3). H2ax was used as the control for γ-H2ax. Values are shown as mean ± SD. **, *P* < 0.01.

To investigate the ability of Hcy to worsen DNA damage, we used MMC to conduct a cellular model of ICLs. After NE4C cells were exposed to MMC in the presence and absence of Hcy for 24 h, the Comet assay was used to detect DNA damage. Our results displayed that only a few comet-positive cells (10.7%) were detected in control NE4C cells (**Fig. 2A and B**). Compared with cells only exposed to MMC (44.0%), supplementing the MMC-treated cell culture medium with Hcy increased the number of damaged cells (60.7%). Consistently, the OTM value of cells co-treated with Hcy and MMC was approximately 1.9-fold higher than that in cells treated only with MMC (*P* < 0.01; **Fig. 2C**).

Phosphorylation of H2AX (γ-H2AX) is the earliest sign of DNA damage. By immunoblot analyses, we found that Hcy did not alter the protein expression of H2ax; however, the γ-H2ax level was significantly increased when NSCs were co-treated with Hcy and MMC (**Fig. 2D and E**). Together, these data suggest that Hcy contributes to the accumulation of DNA damage following treatment with MMC.

### Hcy inhibits monoubiquitination of Fancdi and Fancd2 in murine NSCs upon MMC treatment

**Figure 3:**
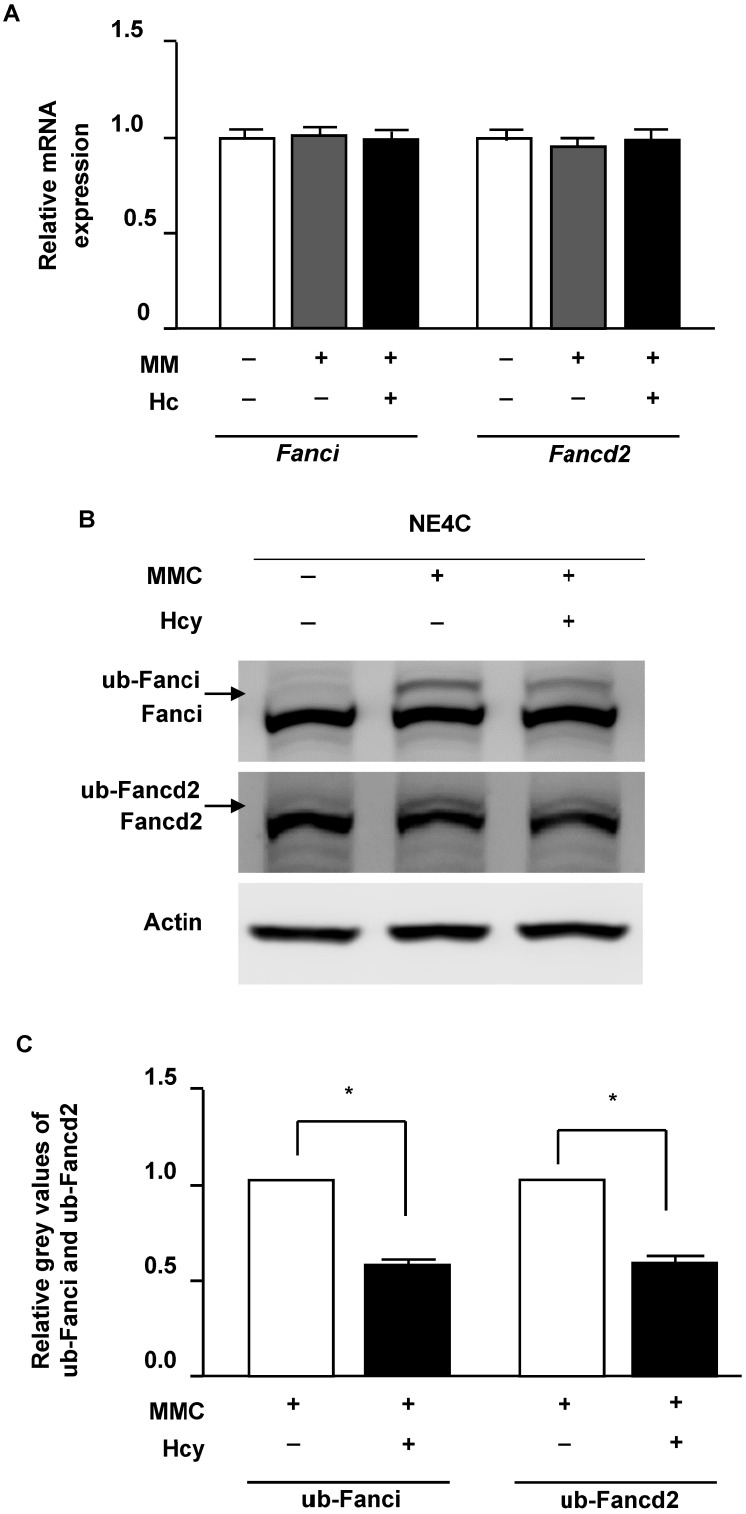
** Hcy inhibits monoubiquitination of Fanci and Fancd2 in NSCs upon MMC treatment.** (**A**) Relative mRNA levels of *Fanci* and *Fancd2*. (**B**) Cells were lysed and used in western blot (WB) analysis using anti-Fanci (lane 1), anti-Fancd2 (lane 2) and anti-Actin (lane 3) antibody. ub-Fanci, Fanci monoubiquitination; ub-Fancd2, Fancd2 monoubiquitination. (**C**) Representative WB statistical results of ub-Fanci and ub-Fancd2. Ub-Fanci and ub-Fancd2 were quantified by densitometry and normalized to Fanci and Fancd2, respectively. All data are shown as mean ± SD of three independent experiments. *, *P* < 0.05.

Following DNA damage of ICLs, FANCI and FANCD2 proteins load on the altered chromatin and initiate repair. We therefore surveyed the effects of Hcy on Fanci and Fancd2 in murine NSCs in the presence of ICLs. Our results showed that neither the mRNA and protein levels of Fanci and Fancd2 were not altered by Hcy when cells were exposed to MMC (**Fig. 3A and B**). In the control group which cells were normal without DNA damage, monoubiquitination levels of Fancd2 and Fanci were low. MMC treatment remarkably increased the levels of ub-Fanci and ub-Fancd2. However, compared with treatment with MMC alone, the ub-Fanci and ub-Fancd2 levels were decreased up to about 60.3% and 55.7% by co-treatment with Hcy and MMC, respectively (**Fig. 3B**).

### Hcy inhibits the Fancd2/Brca1 pathway in murine NSCs upon MMC treatment

**Figure 4:**
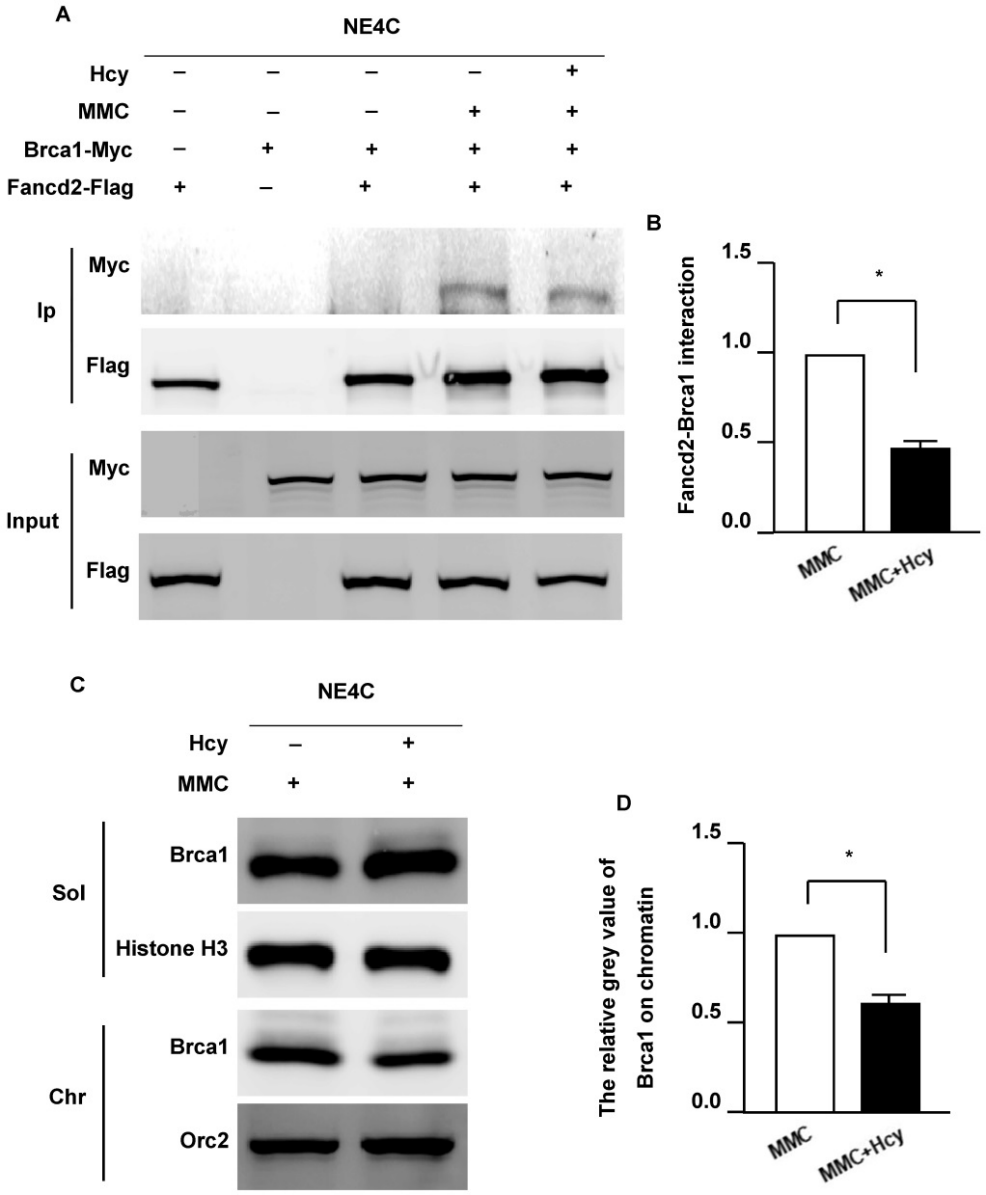
** Hcy inhibits the Fanconi anemia (FA)/Brca1 pathway in murine neural stem cells upon MMC treatment.** (**A**) NSCs were co-transfected with exogenous Fancd2-Flag and Brca1-Myc for 48 h. Brca1 binding to Flag-beads purified Fancd2 was bolted with anti-Myc antibody. Ip, immunoprecipitation. (**B**) Representative WB statistical results of the Fancd2-Brca1 interaction. (**C**) NSCs were subjected to chromatin fraction after treatment. (**D**) Representative WB statistical results of Brca1 on chromatin. Brca1 and Orc2 in the indicated fractions were detected by immunoblotting. Sol, soluble fraction; Chr, chromatin fraction. Histone H3 and Orc2 served as the controls for the soluble fractions and chromatin fractions, respectively. All data are shown as mean ± SD of three independent experiments. *, *P* < 0.05.

FANCD2 monoubiquitination plays a critical role in recruiting BRCA1, FNACJ, and FANCN to form damage-induced foci on chromatin (33). To determine whether Hcy interferes with the Fancd2/Brca1 pathway, we detected the Fancd2-Brca1 interaction by co-overexpressing exogenous Fancd2-Flag and Brca1-Myc in NE4C cells. The interaction between Fancd2 and Brca1 could not be detected in the control group without DNA damage (**Fig. 4A**). Following MMC exposure, the Fancd2-Brca1 interaction was dramatically elevated; however, supplementing the culture medium with Hcy diminished the interaction of Fancd2-Brca1 up to 44.1% (**Fig. 4B**). The weaker Fancd2-Brca1 interaction represents fewer Fancd2-Brca1 complexes on chromatin, suggesting that Hcy disturbs the formation of the Fancd2-Brca1 complex under MMC treatment. These results indicate that Hcy may interrupt the ability of Fancd2 to recruit Brca1 on chromatin. To further investigate whether the association of Brca1 and chromatin was altered, we examined Brca1 by cell fractionation and immunoblotting following MMC exposure with or without Hcy (**Fig. 4C**). After co-treatment with MMC and Hcy, a decreased amount of Brca1 was detected on chromatin (**Fig. 4C and D**). Taken together, the decreased Fancd2-Brca1 interaction and the amount of Brca1 on chromatin represent that the Fancd2/Brca1 pathway is impaired by Hcy in the presence of MMC-induced DNA damage.

## Discussion

As a DNA damage agent, Hcy is associated with neurogenesis during development and adult periods, yet its effect on the DDR is unclear. In the current study, we used murine NCSs to examine whether Hcy worsens DNA damage following MMC treatment, and characterized its possible mechanism of action. The results demonstrated that Hcy accumulated DNA damage and promoted apoptosis following MMC treatment. The FANCD2/BRCA1 pathway is activated following exposure to MMC 40. Notably, compared with exposure to MMC alone, co-exposure to MMC and Hcy diminished the monoubiquitination levels of Fanci and Fancd2, the Fancd2-Brca1 interaction, and the amount of Brca1 on chromatin. These data suggest that Hcy may aggravate DNA damage and promote hypersensitivity to MMC by impairing the Fancd2/Brca1 pathway in murine NSCs under DNA damage.

HHcy, a medical condition of elevated Hcy concentration in the plasma, usually defined as > 15 μM 3, is recognized as a biomarker of several cardiovascular and neurological disorders 8. HHcy can be caused by a high Met and/or low methyl-donor diet in animal models 4,41. Recently, researchers showed that Hcy administration by tail vein injection could also induce HHcy in rats 42. Neurons are much more vulnerable to Hcy than other cell types, such as vascular endothelial cells or astrocytes 11. A concentration of Hcy as low as 0.5 μM can cause apoptosis in cultured hippocampal neurons and it is particularly effective in sensitizing neurons to excitotoxicity, both in cell culture and *in vivo*; while Hcy concentrations up to 10 mM could not kill vascular endothelial cells or astrocytes during a 48 hours exposure period 11. These results suggest that the cytotoxic concentration of Hcy depends on the cell type. The concentration range of Hcy treatment is 0.03-1 mM in cultured neural stem cells 6,43,44. Zhang *et al.* 6 treated NE4C cells with 0.1, 0.5, and 1 mM Hcy, and found that the pathological effects of Hcy were in a dose-dependent manner. In our previous study of DNA damage model induced by hydroxyurea, we have observed that supplementing the cell-culture media with 0.5 mM Hcy aggravated the number of damaged cells in HCT116 cells 35. Therefore, we used 0.5 mM Hcy to observe its effects on accumulation of DNA damage and promotion of cytotoxicity in NE4C cells under conditions of MMC-induced DNA damage in this study.

The extended predisposition of the CNS to Hcy during the embryonic development or in mature stages may be explained by accumulated DNA damage 15,45,46. Hcy is rapidly taken up by neurons through a specific transporter and then accumulates in cells at relatively high concentrations 47. Elevated cellular Hcy levels may induce several kinds of DNA damage including DNA strand breaks, ICLs and mutation 10. And DNA damage may be an early event required for subsequent Hcy-induced oxidative stress in neurons 11. In this study, compared with cells exposed to MMC alone, we found that the percentage of survival cells was approximately 1.5-fold less in cells co-exposed to MMC and Hcy. However, the Comet Assay showed that the percentage of damaged cells and the OTM value of cells co-treated with Hcy and MMC were about 1.4- and 1.9-fold higher, respectively, in cells only treated with MMC. These data suggest that Hcy may inhibit cell viability and exacerbate ICLs induced by MMC. Consistent with our results, Kruman *et al.* 15 have been demonstrated that Hcy could increase cellular DNA strand breaks and aggravate cell death induced by Aβ in hippocampal neurons and a murine model of Alzheimer's disease. In a rat model of focal ischemia, the authors also observed that HHcy aggravated cortex damage after ischemia-reperfusion by inducing autophagosome accumulation and promoting oxidative DNA damage 42. Thus, we speculate that Hcy renders neural cells vulnerable to death by accumulating DNA damage.

The FA pathway is an important DDR pathway for the removal of ICLs. At least 11 complementation groups for FA have been identified, the eight known FA proteins cooperate in a common pathway. Disruption of this pathway results in the weakened repair function of ICLs and can cause clinical abnormalities. The majority of FA patients have congenital neurological disorders, including microcephaly and microphthalmia. Loss of *Fancd2* in mice and zebrafish induced brain developmental abnormalities that have also been reported in FA patients 27,48. In the Fanconi anemia complementation group A (Fanca) and Fanconi anemia complementation group G (Fancg) deficient mice, whose Fancd2 activation and FA/Brca1 pathway functionality are abolished, the neuron number is reduced at the dorsal telencephalon 32,49. The decrease in neuron production is due to an increased apoptosis of neural progenitor cells in embryonic cortices, which can be attributed to DDR defects 33. This evidence implies that the FA pathway plays a pivotal role in the DDR during neurogenesis. Here, we found that Hcy supplement not only inhibits the monoubiquitination levels of Fanci and Fancd2, but also decreases the interaction of Fancd2-Brca1 and Brca1 on the chromosome upon MMC treatment. Given the interaction of FANCD2-Ub and BRCA1 to chromosome suggests that the FA pathway may function to repair damage 29,30; thus, Hcy may inactivate the FA pathway in the presence of DNA damage.

A new mechanism, “protein N-homocysteinylation” (Hcy-N-protein), has recently emerged and is considered to be the major cause of cardiovascular and neurological degenerative disorders in humans 4,50. Plasma Hcy-N-protein is positively related to plasma Hcy. Hcy-thiolactone is a metabolite of Hcy that reacts with protein lysine residues to form Hcy-N-protein, which significantly contributes to Hcy cytotoxicity. N-homocysteinylation is deleterious to protein structure, interaction and function 51. HHcy also appears to exacerbate the effects of other factors implicated in Alzheimer's disease pathology, such as increasing N-homocysteinylated levels of tau and Aβ 4,21. Thus, the inhibitory effect of Hcy on monoubiquitination of Fanci and Fancd2 in our study may be attributed to N-homocysteinylation. The pathological links between N-homocysteinylation and the activities of Fanci and Fancd2 remain to be determined.

Our study has several limitations. First, we conducted experiments in NE4C murine NSCs. It is not clear that if Hcy accumulates DNA damage in any other neural cell types or *in vivo*. And NE4C are neural stem cells with high proliferation capacity (and thus high rate of DNA damage), therefore the results of this study are not easily translatable to the adult human neural tissue (with low proliferative capacity). Second, this study suggests that Hcy may exacerbate DNA damage by disturbing the FA/Brca1 pathway. The monoubiquitination of FANCI and FANCD2 is regulated by Ataxia telangiectasia and Rad3-related protein (ATR) which is an essential regulator of genome integrity. Thus, whether Hcy interferes with the FA pathway by inhibiting ATR activation remains to be uncovered.

## Conclusion

Our study demonstrated that Hcy aggravated DNA damage and promoted death when murine NSCs underwent DNA damage; moreover, Hcy affected the monoubiquitination of Fanci and Fancd2, disturbed the Fancd2-Brca1 interaction, and subsequently reduced Brca1 on chromatin. These data suggest that Hcy can enhance cell death, which contributes to the accumulation of DNA damage and promotion of hypersensitivity to cytotoxicity by impairing the FA/Brca1 pathway in the presence of DNA damage in murine NSCs.

## Figures and Tables

**Table 1:** Primers of qRT-PCR

Genes	Primers	Sequences
*Fanci*	Forward	5'-TGTGGTGAATTTGAGAACGGC-3'
	Reverse	5'-AAGAGGCATCTTCTGGAGTCACTT-3'
*Fancd2*	Forward	5'-GTCAGAGCGCCGTTCTTCTA-3'
	Reverse	5'-AAGGGGCATGGTTTTGGAGG-3'
*Gapdh*	Forward	5'- AGCAGTCCCGTACACTGGCAAAC-3'
	Reverse	5'- TCTGTGGTGATGTAAATGTCCTCT-3'
